# Metagenomic Analysis of Viromes of *Aedes* Mosquitoes across India

**DOI:** 10.3390/v16010109

**Published:** 2024-01-12

**Authors:** Abhranil Gangopadhayya, Kavita Lole, Onkar Ghuge, Ashwini Ramdasi, Asmita Kamble, Diya Roy, Shivani Thakar, Amol Nath, AB Sudeep, Sarah Cherian

**Affiliations:** 1 Hepatitis Group, Indian Council of Medical Research-National Institute of Virology, Microbial Containment Complex, Sus Road, Pashan, Pune 411021, India; agangopadhayya95@gmail.com (A.G.); lole.k@gov.in (K.L.); onkarghuge1506@gmail.com (O.G.); ashwiniramdasi76@gmail.com (A.R.); shivanithakar.08@gmail.com (S.T.); anniv271@gmail.com (A.N.); 2 Bioinformatics and Data Management Group, Indian Council of Medical Research-National Institute of Virology, 20-A, Dr. Ambedkar Road, Pune 411001, India; asmitakamble13@gmail.com (A.K.); diyaroyubkv@gmail.com (D.R.)

**Keywords:** Virome, *Aedes aegypti*, Aedes albopictus, PCLV, CFAV, CfGV

## Abstract

Metagenomic analysis of *Aedes aegypti* and *Ae. albopictus* mosquitoes from diverse geographical regions of India revealed the presence of several insect viruses of human interest. Most abundant reads found in *Ae. aegypti* mosquitoes were of Phasi Charoen-like virus (PCLV), *Choristoneura fumiferana* granulovirus (CfGV), Cell fusing agent virus (CFAV), and Wenzhou sobemo-like virus 4 (WSLV4), whereas WSLV4 and CfGV constituted the highest percentage of reads in *Ae. albopictus* viromes. Other reads that were of low percentage included Hubei mosquito virus 2 (HMV2), Porcine astrovirus 4 (PAstV4), and Wild Boar astrovirus (WBAstV). PCLV and CFAV, which were found to be abundant in *Ae*. *aegypti* viromes were absent in *Ae. albopictus* viromes. Among the viromes analyzed, *Ae. aegypti* sampled from Pune showed the highest percentage (79.82%) of viral reads, while *Ae*. *aegypti* mosquitoes sampled from Dibrugarh showed the lowest percentage (3.47%). Shamonda orthobunyavirus (SHAV), African swine fever virus (ASFV), Aroa virus (AROAV), and Ilheus virus (ILHV), having the potential to infect vertebrates, including humans, were also detected in both mosquito species, albeit with low read numbers. Reads of gemykibivirus, avian retrovirus, bacteriophages, herpesviruses, and viruses infecting protozoans, algae, etc., were also detected in the mosquitoes. A high percentage of reads in the *Ae. albopictus* mosquito samples belonged to unclassified viruses and warrant further investigation. The data generated in the present work may not only lead to studies to explain the influence of these viruses on the replication and transmission of viruses of clinical importance but also to find applications as biocontrol agents against pathogenic viruses.

## 1. Introduction

Arboviral diseases continue to plague human populations around the world, especially in tropical and subtropical countries. Mosquito-borne viruses, i.e., dengue virus (DENV), Zika virus (ZIKV), West Nile virus (WNV), Japanese encephalitis virus (JEV), Chikungunya virus (CHIKV), and tick-borne viruses, such as Crimean Congo hemorrhagic fever virus (CCHFV), Tick-borne encephalitis virus (TBEV), etc., contribute to the main burden. *Aedes aegypti*, the day-biting mosquito, has emerged as a major concern in the last few decades as it transmits deadly viruses, i.e., DENV, CHIKV, Yellow fever virus, (YFV) and ZIKV [1], which have tremendous public health importance. It is estimated that half the global population is under the threat of dengue, and ~390 million people contract DENV infection annually, of which 96 million develop symptomatic disease [2].

Mosquitoes also harbor certain viruses that replicate within select arthropod host species, thus earning them the name “insect-specific viruses” (ISVs), which have the potential to modulate the replication of arboviruses of human importance. Nhumirim virus, an insect-specific flavivirus (ISF), was not only found to suppress replication of DENV serotype-2 (DENV-2) and ZIKV in *Aedes albopictus* C6/36 cells but also to affect vector competence of *Ae. aegypti* mosquitoes to DENV-2 and ZIKV [3]. Growing evidence suggests that ISVs may have been the ancestors of currently circulating human-infecting arboviruses, as proven by *Alphaviruses* belonging to the Western equine encephalitis virus complex, *viz.*, Eilat virus (EILV) and Taï Forest alphavirus (TALV) [4]. A genetic surveillance of flavivirus-specific sequences from *Culex tritaeniorhynchus* mosquitoes in Assam, India, revealed the presence of an ISV with the highest sequence identity to the Palm Creek virus [5]. Novel control measures to reduce virus transmission are emerging, including usage of the *Ae. aegypti* endosymbiont *Wolbachia pipientis*. A cluster-randomized trial using *Wolbachia*-infected *Ae. aegypti* introgression into natural mosquito populations showed a significant decrease in dengue incidence and hospitalizations [6]. ISVs, similar to *Wolbachia*, are speculated to have the potential to be used as biocontrol agents [7].

Metagenomics is an advancing field that involves the genetic sampling of micro-organisms to gain insight into their composition and diversity [8]. This reveals a plethora of organisms present in any microbial community in various environmental and clinical samples. The virome is the total of all the virus-specific sequences discovered in a sample, designated to their respective taxonomic virus units. Analysis of viromes, as opposed to total metagenomes, has revealed a greater richness in viral Operational Taxonomic Units (vOTUs), and this approach can thus reveal vastly diverse viruses that exist in any kind of sample [9]. Virome analysis of hematophagous arthropods (mosquitoes) is a lucrative approach to identifying the presence of viruses of interest, which often consist of human pathogens and ISVs. Virome analysis may reveal human pathogens that may have been overlooked by surveillance measures and also discover viruses that might evolve into potential human pathogens [10]. It has been reported that *Ae. aegypti* and *Cx. quinquefasciatus* mosquitoes harbor core viromes that are distinct in diversity, with the former having a richer eukaryotic virome and the latter having a more diverse variety of phages [11]. Metaviromic analysis can employ the use of amplification to enhance the detection of different viral species when random anchored primers are used to amplify parts of viral genomes in a sequence-independent manner [12].

In a previous study, we reported an abundance of Phasi Charoen-like phasivirus (PCLV) in *Ae. aegypti* populations throughout India, a country endemic to DENV and CHIKV [13]. Here, we report the metagenomic analysis of virome constituents of *Ae. aegypti* and *Ae. albopictus* mosquitoes sampled from geographically diverse regions of India.

## 2. Materials and Methods

### 2.1. Collection and Storage of Wild Mosquitoes, Larvae and Pupae

**Figure 1:**
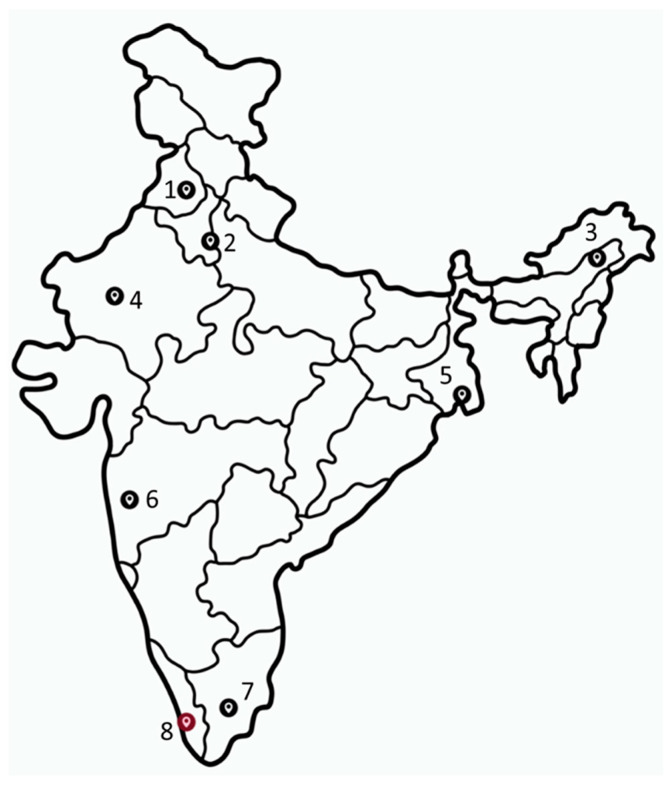
Map of India showing collection sites. Red indicates the site for only the *Ae. albopictus* collection. 1: Patiala, Punjab; 2: New Delhi; 3: Dibrugarh, Assam; 4: Jodhpur, Rajasthan; 5: Kolkata, West Bengal; 6: Pune, Maharashtra, 7: Madurai, Tamil Nadu, 8: Alappuzha, Kerala. Map source: vecteezy.com (accessed on 15 October 2023).

*Ae. aegypti* and *Ae. albopictus* mosquitoes were collected from their natural breeding habitats and resting spots across various locations (Figure 1) using handheld-manual aspirators and brought to the Medical Entomology and Zoology Group, Indian Council of Medical Research—National Institute of Virology (ICMR-NIV), Pune. *Ae. albopictus* mosquitoes from Alappuzha, Kerala, were collected and sent by the ICMR-NIV Kerala unit through a consistent cold chain. Adults were identified using entomological keys, sorted by species and sex, pooled (n = 20), and stored at −80 °C for further use. Larvae and pupae were reared to adults and processed similarly.

### 2.2. Viral RNA Extraction, Double-Stranded Complementary DNA (cDNA) Preparation, and Sequence-Independent Single Primer Amplification (SISPA)

Pools of 20 adult female mosquitoes were processed for sequencing library preparation according to a previously published protocol [13], with a few modifications. In brief, mosquitoes were washed with sterile MilliQ water and homogenized in liquid nitrogen using a sterile mortar and pestle. Homogenized tissue was resuspended in 1X phosphate-buffered saline (PBS) containing 5 millimolar (mM) magnesium chloride (MgCl_2_) and 1.4 mM Dithiothreitol (DTT) (Invitrogen) and centrifuged at 17,000× *g* for 5 min at 4 °C. The supernatant was filtered through a 0.22 µm syringe filter (Merck Millipore, Burlington, MA, USA) and treated with 20 Units (U) each of TURBO DNase and RNase I (Thermo Scientific, Waltham, MA, USA) for 1 h. Viral RNA was extracted using the QIAamp Viral RNA Mini kit (Qiagen, Hilden, Germany). The eluted RNA was quantified using a Qubit RNA HS assay kit and Qubit Flex Fluorometer (Invitrogen, Waltham, MA, USA). A maximum of 100 nanograms (ng) RNA was used for first-strand cDNA synthesis, followed by RNaseH treatment. Second-strand synthesis using Klenow fragment and Sequence-independent single-primer amplification (SISPA) [14] were carried out as described earlier [13]. The primers used for SISPA are given in Table 1. Three separate PCR products were generated for each sample using P5, P12, and IDT-K sets of primers. The resulting amplified double-stranded cDNA (ds-cDNA) was purified using AMPure XP Reagent magnetic beads (Beckman Coulter, Brea, CA, USA).

### 2.3. Library Preparation and Sequencing on Oxford Nanopore MinION Mk1C Sequencer

Purified amplicons were used to prepare sequencing libraries using the Ligation Sequencing Kit, SQK-LSK109, and the Native Barcoding Kit, EXP-NBD104 (Oxford Nanopore Technologies (ONT), UK), according to the manufacturer’s protocol. Briefly, 200 femtomoles (fmol) of the P5, P12, and IDT-K SISPA reaction products were subjected to DNA repair and end preparation using NEBNext Ultra II End repair/dA-tailing Module (New England Biolabs, Ipswich, MA, USA). Equimolar amounts of the end-prepped DNA were subjected to barcoding using Native Barcoding Kit EXP-NBD104 and NEB Blunt/TA Ligase Master Mix (New England Biolabs, Ipswich, MA, USA). The barcoded samples were pooled together, and adapter ligation was carried out using the adapter mix and the NEBNext Quick Ligation Module (New England Biolabs, Ipswich, MA, USA). At each level, i.e., DNA repair/end preparation, native barcoding, pooling of barcoded samples, and adaptor ligation, purification was carried out using AMPure XP Reagent magnetic beads (Beckman Coulter, Brea, CA, USA), and quantitation using Qubit dsDNA HS assay kit and Qubit Flex fluorometer (Invitrogen, Waltham, MA, USA). A final amount of 100–200 fmol of the adaptor-ligated library was loaded for sequencing onto a SpotON FLO-MIN106D flow cell (version R9.4.1) set on an ONT MinION Mk1C device (Oxford, UK), with the MinKNOW interface. For *Ae. albopictus* samples, a final amount of 20 fmol was loaded onto a SpotON FLO-MIN111 flow cell (version R10.4) set on the Mk1C device. Basecalling was performed in real-time using the Fast Basecalling option. Data acquisition was performed with a minimum q-score cutoff of 8. The minimum read length was set at 200 bases. Demultiplexed and quality-controlled reads were stored in output files that were acquired in FASTQ format without compression. A maximum of 5000 reads per file was permitted, and each run was carried out for at least 14 h.

### 2.4. Analysis of Raw Metagenomics Data

For each barcode, the FASTQ files containing raw reads were merged using Commander-NGS software (Genotypic Technology Pvt. Ltd., Bengaluru, India) and processed using Porechop v0.2.4 software (https://github.com/rrwick/Porechop (accessed on 30 May 2022)) for removal of adapter sequences. Trim Galore! v0.6.4_dev (https://github.com/FelixKrueger/TrimGalore (accessed on 30 May 2022)) was used to trim primers from merged FASTQ files and further analyzed using EPI2ME Portal (https://epi2me.nanoporetech.com (accessed on 22 July 2022)), a cloud-based algorithm including analytical workflow for taxonomic classification (ONT). All the viral species identified through EPI2ME that were given “no rank” through the classification pipeline were checked through the “Commander-NGS” software for the authenticity of read classification. The merged FASTQ file for each sample was processed through the Long Reads Variant Calling pipeline of the “Commander-NGS” software using RefSeq whole genome sequences for each virus species obtained through EPI2ME. This pipeline follows minimap2, Samtools, and bcftools. Viral species that failed to generate any results through this pipeline were excluded from the analysis. Percent genome coverage for each virus was calculated using Samtools. Information on the families and known hosts of the viruses was obtained through the National Centre for Biotechnology Information (NCBI) Taxonomy Browser [15] and the Virus-Host DB [16]. To obtain consensus FASTA files of whole genome sequences, the merged FASTQ file representing metagenomics data of each location was uploaded to the Genome Detective online server for analysis by its Virus Tool version 2.49 [17]. Upon report generation, each virus detected by EPI2ME and with >50% genome coverage was chosen, and the corresponding FASTA files were downloaded for further analyses.

### 2.5. Sanger Sequencing to Obtain Complete Cell Fusing Agent Virus (CFAV) Genome Sequence

To obtain the complete genome sequence of CFAV, Reverse Transcription-PCR (RT-PCR) followed by Sanger sequencing was carried out for the 5′ end of the genome. Primers specific for the first ~550 bases of the CFAV genome were designed (Table 1) using alignment of CFAV whole genome reference sequences from across the world [18]. Primer specificity was checked using the Basic Local Alignment Search Tool (BLAST, NCBI). Melting temperatures (T_m_) and annealing temperature for the primers were checked using the Thermo Fisher Scientific T_m_ Calculator (https://www.thermofisher.com/in/en/home/brands/thermo-scientific/molecular-biology/molecular-biology-learning-center/molecular-biology-resource-library/thermo-scientific-web-tools/tm-calculator.html (accessed on 12 August 2022)). Mosquitoes were homogenized in Minimum Essential Medium (HiMedia, Thane, India), centrifuged at 17,000× *g* for 5 min at 4 °C, and the supernatant was used for viral RNA extraction using QIAamp Viral RNA Mini kit (Qiagen, Hilden, Germany). Ten microliters (µL) of the extracted RNA was used to perform RT-PCR using SuperScript™ III One-Step RT-PCR System with Platinum™ Taq DNA Polymerase kit (Thermo Scientific, Waltham, MA, USA). Each 50 µL reaction composed of 25.0 µL of 2X Reaction Mix, 50 picomoles each of forward and reverse primers (Table 1), 3.0 µL Nuclease Free Water (NFW), 2.0 µL of the SuperScript III RT/Platinum Taq Mix, and 10 µL of template RNA. The reaction conditions were: 50 °C for 45 min, 95 °C for 10 min, followed by 35 cycles at 94 °C for 1 min, 55 °C for 1 min, and 68 °C for 90 s, and a final extension at 68 °C for 5 min. PCR products were analyzed on 2% agarose gel and purified using the MinElute Gel Extraction Kit (Qiagen, Hilden, Germany). The purified PCR product was sequenced from both strands using Applied Biosystems™ BigDye™ Terminator v3.1 Cycle Sequencing Kit and analyzed using Applied Biosystems™ 3130xl Genetic Analyzer (Thermo Scientific, Waltham, MA, USA).

### 2.6. Phylogenetic Analysis of Metagenomics Derived Whole Genome Sequences of Viruses

Consensus FASTA files were used for phylogenetic analyses using MEGA software, version 5.2. Whole genome reference sequences were downloaded from the NCBI database and aligned using the “Multiple Alignment using Fast Fourier Transform” (MAFFT) online server [18]. For sequences of yet unclassified viruses, BLAST searches were performed to find the most identical nucleotide sequences, which were subsequently used for alignment and analyses. The same was also performed for viruses that gave ambiguous results through BLAST, i.e., showing high identities with sequences of multiple taxa. Phylogenetic tree construction was completed using MEGA v.5.2 according to the best-fit substitution model deduced by the software for each alignment. BLAST searches were performed for the generated whole genomes to find respective sequences showing the highest percent nucleotide identity (PNI). PNIs for the CFAV whole genome sequence were calculated using the Clustal2.1 algorithm on the MAFFT server.

## 3. Results

### 3.1. Length and Count Statistics of Sequencing Data Generated on ONT Platform

The average quality score calculated for all the samples ranged between 10.75 and 12.67 (Table 2), and average read lengths ranged between 280 and 476 base pairs (bp).Viruses with the most abundant reads found in *Ae. aegypti* mosquitoes were PCLV, *Choristoneura fumiferana* granulovirus (CfGV), CFAV, and Wenzhou sobemo-like virus-4 (WSLV4). In *Ae. albopictus*, WSLV4 and CfGV constituted the highest percentage of virome reads and were conspicuous in the absence of PCLV and CFAV.

There appeared to be no correlation between the number of analyzed reads and the final number of classified viral reads, as evidenced by *Ae. aegypti* samples from Dibrugarh, Assam, having 2,566,367 analyzed reads with 38,089 viral reads, and New Delhi having 1,728,868 analyzed reads with 140,179 viral reads (Table 2). Among the *Ae. aegypti* samples from different locations, Pune had the highest percentage of viral reads (79.82%) while Dibrugarh had the lowest percentage (3.47%). *Ae. aegypti* mosquito samples from Jodhpur had the highest percentage of PCLV reads (98.82%), while the mosquito samples from Dibrugarh had the lowest percentage (62.96%). Of the two *Ae. albopictus* viromes analyzed, the mosquito virome of Alappuzha, Kerala, samples had fewer WSLV4 reads. A high percentage of reads in both *Ae. aegypti* and *Ae. albopictus* samples remained unclassified, possibly indicating either an inability of the applied workflow to map the reads or representing potentially uncharacterized organisms.

### 3.2. Virus Taxa Identified through Metaviromics

Table 3 show viral species identified and results for the species-specific reads, which were sorted and mapped against reference genomes from the Centrifuge database through the EPI2ME online server. Reads classified as *Homo sapiens* were the most abundant in viromes of mosquitoes from all locations. There was a wide diversity of viral species in all samples. The mosquito virome belonging to Patiala showed the highest species-wise diversity, while samples collected from Kolkata showed the lowest diversity. There was no correlation between the number of species identified and the region to which each location belonged. Interestingly, Dibrugarh mosquitoes had the maximum number of viral species with high reads of PCLV, CFAV, WSLV4, CfGV, Hubei mosquito virus 2, Porcine astrovirus 4 and Wild Boar astrovirus (Astrovirus wild boar/WBAstV-1/2011/HUN), with the six remaining species having only negligible reads (1–10). The mosquito virome derived from the Kolkata samples appeared to have a few identified taxa at the species level. Similarly, there appeared to be no correlation between the number of viral taxa identified and the sample location.

*Ae. albopictus* mosquito viromes were found to lack the two most prominent viruses found in the viromes of *Ae. aegypti* mosquitoes, i.e., PCLV and CFAV. Otherwise, multiple viruses were found to be common to both species. Apart from WSLV4 and CfGV, Hubei mosquito virus 2 (HMV2) was the most abundant constituent of the two *Ae. albopictus* viromes.

### 3.3. Viruses Identified Have a Wide Range of Hosts

**Figure 2:**
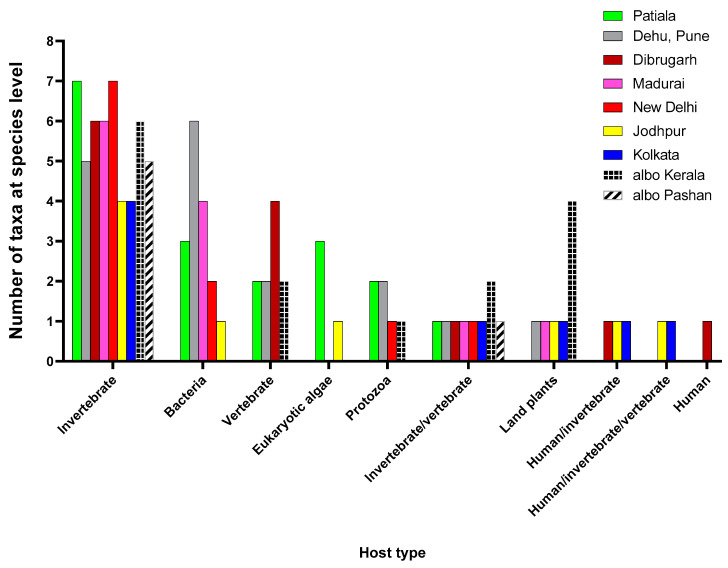
Host distribution for the virus taxa identified for the viromes: The host type information was obtained from the NCBI Taxonomy database and Virus-Host DB, and the number of taxa identified in each virome for each host type was plotted. The graph was plotted using GraphPad Prism v.8.4.2. Wherever it is not mentioned, viromes are from *Ae. aegypti* mosquitoes, while *Ae. albopictus* are mentioned as albo.

The host type showing the most abundance of viral taxa represented invertebrates, indicating a dominance of ISVs (Figure 2). Among the analyzed viromes, mosquitoes collected from Patiala and New Delhi represented the most ISVs. There were also viruses that had both invertebrates and vertebrates, including humans, as hosts. Shamonda orthobunyavirus (SHAV) and African swine fever virus (ASFV), known to infect both invertebrate and vertebrate hosts, were detected in *Ae. albopictus* mosquitoes from Kerala, while Ilheus virus (ILHV), capable of infecting both humans and invertebrates, were found in *Ae. aegypti* mosquitoes obtained from Dibrugarh, Jodhpur and Kolkata, though with few reads. Considering the abundance of RNA viruses identified through our protocol and the low number of reads classified as ILHV, it is unlikely that a viable infectious virus was actually present; therefore, it warrants further studies for confirmation. Similarly, Aroa virus (AROAV), a virus capable of infecting invertebrate and vertebrate hosts was detected in *Ae. aegypti* mosquitoes collected from Jodhpur and Kolkata, albeit with very few read numbers.

Several taxa were identified as having a host range limited only to vertebrates. Four such viruses were found in mosquito samples obtained from Dibrugarh, i.e., Porcine astrovirus 2 (PoAstV2), Porcine astrovirus 4 (PoAstV4), Wild boar astrovirus (WBAstV), and a fish virus, Cyprinid herpesvirus 2 (CyHV-2). Patiala mosquitoes had two species of vertebrate viruses: the avian endogenous retrovirus EAV-HP and the *Rhinolophus* associated gemykibivirus 2. *Ae. albopictus* virome of Kerala had Elephant endotheliotropic herpesvirus-4 and CyHV-2. Interestingly, Astrovirus MLB2, a virus with a host range limited only to humans, was found in the samples of Dibrugarh (<10 reads). However, more studies using RT-PCR and sequencing are needed for confirmation.

In terms of viruses having a bacteria-restricted host range, i.e., bacteriophages, all the *Ae. aegypti* viromes, except for the samples from Dibrugarh and Kolkata, harbored different phage populations. These ranged from six phage species detected in *Ae. aegypti* mosquito samples from Pune to only one from the Jodhpur samples. The phage species in mosquitoes sampled from Pune included *Lactobacillus* virus LP65, *Bacillus* virus G, *Lactobacillus* virus LLKu, *Tenacibaculum* phage PTm1, *Lactococcus* virus KSY1, and *Vibrio* phage Douglas 12A4. *Lactobacillus* virus LP65, *Bacillus* virus G, and *Lactobacillus* virus LLKu phages were also found in mosquitoes sampled from Patiala. Mosquito samples from New Delhi had *Lactobacillus* virus LP65 and *Sphingomonas* phage PAU. The mosquitoes sampled from Madurai harbored a distinct phageome consisting of *Serratia* phage Muldoon, *Synechococcus* phage S-CAM4, *Salinivibrio* phage CW02, and *Mycobacterium* phage UnionJack. Mosquitoes collected from Jodhpur had only *Synechococcus* phage S-CAM22. Thus, phages infecting *Lactobacillus* and *Lactococcus* were found in the viromes of samples from Pune, Patiala, and New Delhi, indicating a possible presence of these bacteria in the mosquitoes. Phages that infect members of the bacterial family *Vibrionaceae* were found in mosquito viromes of Pune and Madurai. Mosquitoes sampled from Madurai also possessed phages infecting bacteria belonging to taxa *Serratia*, *Synechococcus*, and *Mycobacterium*, indicating a possible circulation of these bacteria in the location.

Viruses infecting protozoa were also detected in all the viromes but in negligible numbers (1–10). Other than the giant viruses *Pandoravirus neocaledonia* (Pune) and *P. quercus* (Patiala), Pacmanvirus A23 and *Acanthamoeba polyphaga* mimivirus (both infecting *Acanthamoeba*) were found in Patiala and Pune *Ae. aegypti*, respectively. The Pacmanvirus A23 infects *Acanthamoeba castellani*, which is also a host for viruses of the *Pandoravirus* genus. *P. quercus* was also found in the virome of *Ae. albopictus* mosquitoes from Kerala, whereas Golden Marseillevirus was found in the mosquito virome of *Ae. aegypti* from New Delhi. The presence of giant viruses in the mosquitoes could be from the aquatic habitats of the mosquito larvae that are often shared by their protozoan hosts.

Viruses having eukaryotic algae as hosts were detected in Jodhpur and Patiala mosquitoes, indicating a possible presence of their hosts *Ostreococcus lucimarinus*, *Chrysochromulina ericina*, and *Emiliania huxleyi* in the mosquitoes or their larval/pupal stages.

Mosquito viromes also showed the presence of plant viruses. Kerala *Ae. albopictus* mosquitoes had four taxa belonging to the genus *Tobamovirus*, i.e., Tobacco mosaic virus (TMV), Tobacco mild green mosaic virus (TMGMV), Tomato brown rugose fruit virus, and Tomato mottle mosaic virus. TMGMV and TMV were also detected in Jodhpur and Kolkata mosquito viromes, respectively. Jacquemontia yellow vein virus (JacYVV), a *Begomovirus*, was found in Madurai mosquitoes, while a potyvirus, the Chili ringspot virus (ChiRSV), was found in Pune *Ae. aegypti* mosquitoes, possibly acquired from the nectar of flowers on which male mosquitoes feed and subsequent transmission to their female mating partners.

### 3.4. ISVs Dominate the Viromes of Ae. aegypti and Ae. albopictus

**Figure 3:**
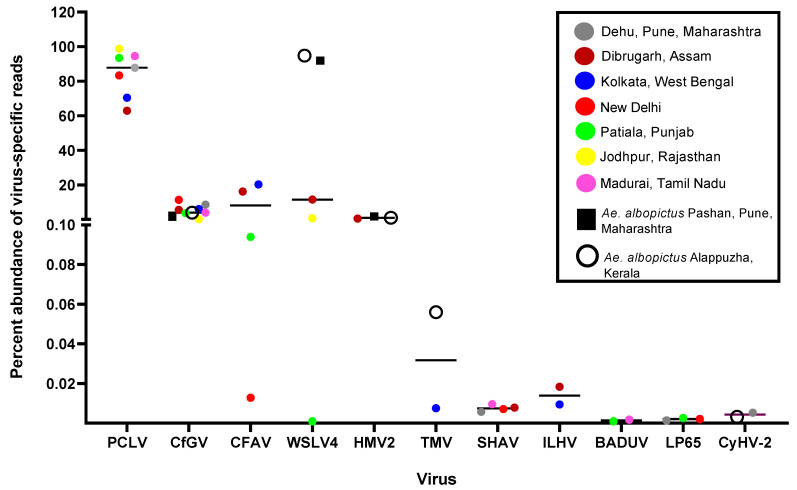
Abundance of reads corresponding to viruses found in *Aedes* viromes: The percentage of reads was calculated with respect to the total viral reads for each virome. Only viruses having more than one read were considered. The graph was plotted using GraphPad Prism v.8.4.2.

PCLV, belonging to the order *Bunyavirales* and family *Phenuiviridae*, represented the highest abundance of reads among all the virus-specific reads in viromes of *Ae. aegypti* across India (Figure 3). PCLV has a three-segmented genome, segment L (NC_038262.1), segment M (NC_038261.1), and segment S (NC_038263.1), corresponding to three Open Reading Frames (ORFs) for RNA-dependent RNA polymerase, glycoprotein, and nucleocapsid, respectively. Jodhpur mosquitoes had the highest abundance (98.82%), Dibrugarh had the lowest (62.96%), and *Ae. albopictus* completely lacked PCLV reads. The *Orthobunyavirus* SHAV also has a three-segmented genome. Similarly, Badu phasivirus (BADUV; order *Bunyavirales*) possesses three genomic segments similar to PCLV and SHAV. WSLV4 had the highest abundance of comparative reads in Alappuzha and Pune *Ae. albopictus* viromes (Figure 3).

CFAV reads were found to be the most abundant in Dibrugarh (16.17%) and Kolkata (20.25%) and the lowest in New Delhi (0.01%) *Ae. aegypti* mosquitoes. However, its abundance remained less than that of PCLV. CfGV (a betabaculovirus) showed an almost comparable percent abundance in viromes of both *Ae. aegypti* and *Ae. albopictus*, with an average value of 4.97%. Mosquito samples from New Delhi showed the highest average value (11.35%), whereas Jodhpur had the lowest (0.12%) abundance of CfGV reads. Despite having the highest abundance, we could not generate a consensus genomic sequence of >50% coverage, probably due to its relatively large virus genome (approximately 104,000 bp). BLAST analysis of generated CfGV sequences from this study showed the highest nucleotide identity (99.97%) with CfGV reference sequence (NC_008168.1) from the USA. Overall, the viruses with the most abundant reads among viromes had invertebrates as their specific hosts, including PCLV, CfGV, CFAV, WSLV4, and HMV2, suggestive of a dominance of ISVs in the mosquito populations of India. Three (CfGV, WSLV4,and HMV2) out of the five viral species were found commonly in both the *Aedes* species. Reads corresponding to viruses of vertebrate host, i.e., SHAV, ILHV, and CyHV-2, were very low, and hence, the genomes of these viruses could not be assembled (Figure 3).

### 3.5. Read Depths and Genome Coverage

**Figure 4:**
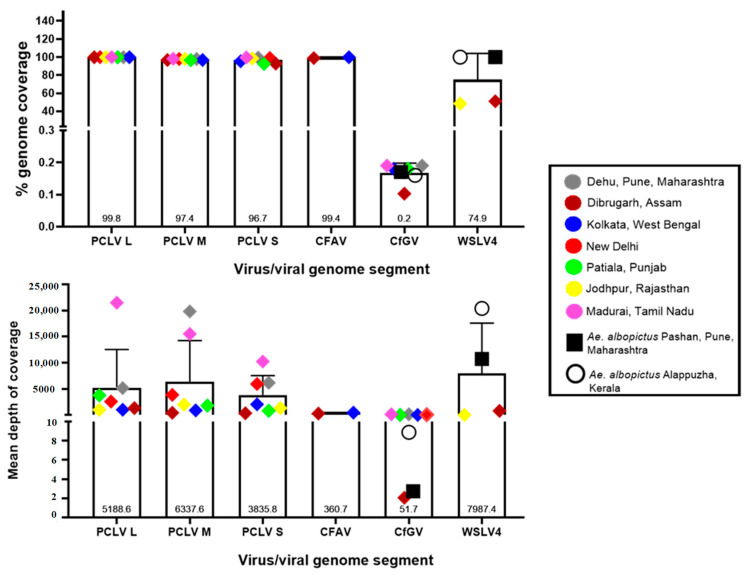
Percent sequence coverage and mean depth for viruses with most abundant reads: Coverage percentage and mean depth were calculated using Samtools. The graphs were plotted using GraphPad Prism v.8.4.2.

Figure 4 depicts the percent genomic coverage and depth, i.e., the number of times the entire genome or sequenced part of the genome was covered in the analyzed viromes. Nearly full genome coverage was achieved for PCLV from the sequences generated in this study, with an average of 96.7%, 97.4%, and 99.8% for L, M, and S segments, respectively. While the coverage values were relatively uniform throughout the analyzed viromes, there were intra-location and intra-segment variations in the depths of coverage, i.e., the L segment of PCLV had a read depth varying from 982 in the Jodhpur mosquito virome to 21,494.6 in the Madurai virome. Similarly, the Pune mosquito virome yielded a depth of 5149.21, 6159.9, and 19840.7 for segments L, S, and M, respectively. The varying trend of read depth may be due to the varying efficiency of the SISPA protocol. For CfGV, despite being present in all analyzed *Aedes* viromes, genomic coverage was almost negligible (0.2% on average), albeit with an average depth of sequence coverage of 51.7 (with Dibrugarh and Pune *Ae. albopictus* mosquitoes showing the lowest depth values). WSLV4 interestingly had an almost complete genome coverage in *Ae. albopictus* virome data, with Pune and Kerala having depths of 10,732 and 20,405, respectively. Jodhpur and Dibrugarh *Ae. aegypti* viromes showing hits of WSLV4 had depths of coverage of 11.08 and 801.22, respectively. For CFAV, mosquitoes from Kolkata and Dibrugarh showed genome coverage depth of 443.69 and 277.78, respectively, with nearly 100% coverage for genomes. For New Delhi and Patiala mosquitoes, it was not possible to derive a complete consensus genomic sequence.

### 3.6. Aedes albopictus Viromes Were Dominated by Unclassified Viruses

WSLV4 and HMV2 are both unclassified members of the realm *Riboviria*, which includes viruses possessing an RNA genome. Due to WSLV4 and HMV2 constituting a large proportion of the reads in both the *Ae. albopictus* viromes, we observed a dominance of unclassified virus families in terms of viral reads. The next most dominant virus family is *Baculoviridae*, owing to the dominance of CfGV reads in these viromes. *Virgaviridae* is constituted by viruses of the genus *Tobamovirus* in the Kerala *Ae. albopictus* virome and has a notable percentage due to the total number of reads from all its members.

**Figure 5:**
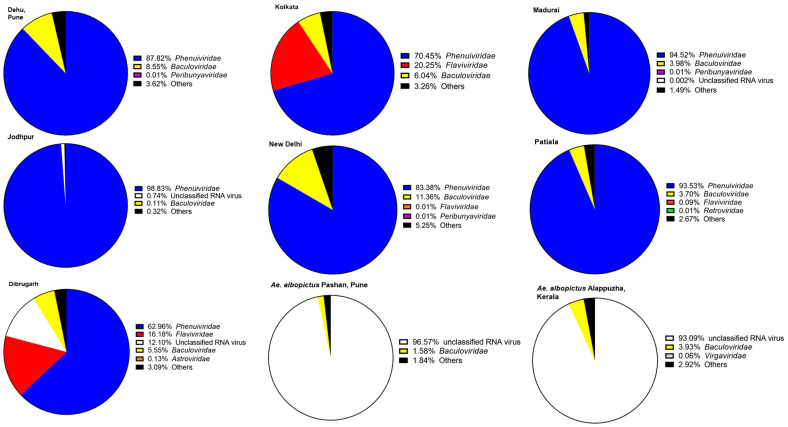
Distribution for the taxa identified in all viromes: Only viruses that had more than 10 reads were considered for assigning taxa. The remaining are referred to as ‘Others’. Information on virus families was obtained from the NCBI Taxonomy database. The graphs were plotted using GraphPad Prism v.8.4.2.

Members of the family *Phenuiviridae* dominated the *Ae. aegypti* viromes from all the locations (Figure 5), and its abundance varied from 62.96% (Dibrugarh) to 98.83% (Jodhpur). The major contributors to this family included PCLV and BADUV, among others. SHAV belonging to the Peribunyaviridae family contributed to the reads in the viromes of *Ae. aegypti* from Pune, Madurai, and Delhi. A large number of reads matching CfGV was present in all the analyzed viromes. Reads representing Avian endogenous retrovirus EAV-HP (Family: *Retroviridae*) were found in Patiala mosquitoes, indicating the feeding of mosquitoes on avian species. Members of the family *Astroviridae*, PoAstV4, and the WBAstV were detected in Dibrugarh *Ae. aegypti* mosquitoes. High numbers of reads of both viruses enabled the generation of consensus genomes with considerable coverage. These are unclassified mamastroviruses of the genus *Mamastrovirus*. Family *Flaviviridae* had viral read contributions from several species. CFAV and ILHV were detected in Kolkata and Dibrugarh mosquitoes, while AROAV was found in Kolkata mosquitoes. Overall, Dibrugarh *Ae. aegypti* mosquitoes were found to possess the highest family-level diversity of viruses; however, a comparatively low percentage of *Phenuiviridae* reads in these mosquitoes indicated competition among the viruses.

It appeared that *Ae. albopictus* mosquitoes had a far higher percentage of unclassified viruses than *Ae. aegypti* mosquitoes. Pune *Ae*. *albopictus* mosquitoes had a marginally higher percentage of unclassified viruses (96.57%) than Alappuzha mosquitoes (93.09%). Unclassified viruses contribute to the sequences of viruses that have not yet been assigned to any taxon. One can observe a uniform presence of such viruses throughout all the viromes described here, with read numbers greater than 10.

**Figure 6:**
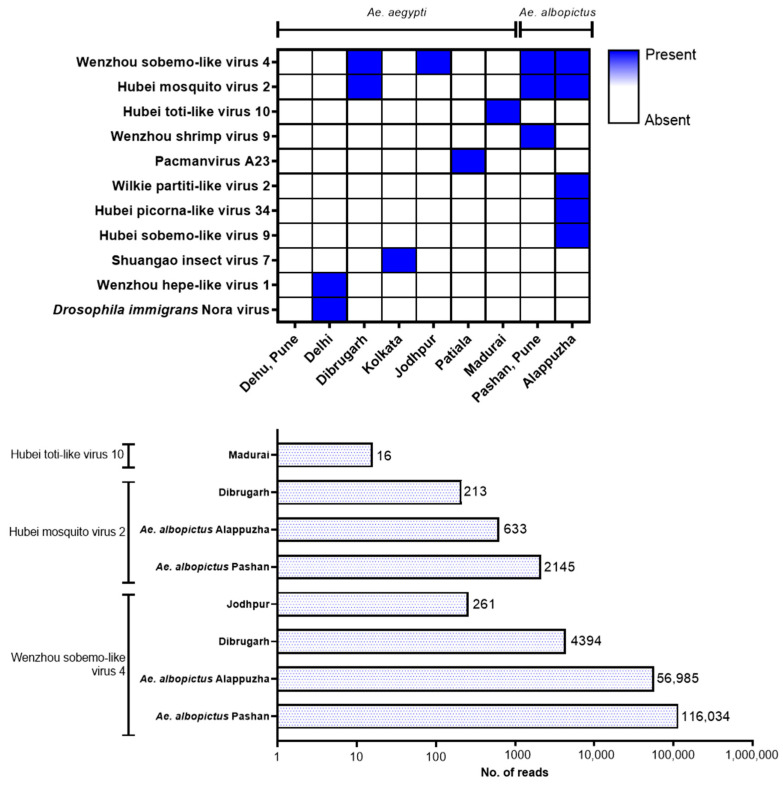
Unclassified viruses: Heatmap showing distribution with respect to the location of mosquitoes (**top**), number of reads corresponding to each identified unclassified virus that had >10 reads in any virome (**bottom**). The graphs were plotted using GraphPad Prism v.8.4.2.

Details of unclassified viruses discovered in this study are given in Figure 6. Kerala *Ae. albopictus* mosquito virome displayed a wide variety of unclassified viruses, with hosts including mosquitoes (WSLV4, HMV2, and Wilkie partiti-like virus-2), beetles (Hubei picorna-like virus-34), and spiders (Hubei sobemo-like virus-9) while no unclassified viruses were detected in the virome of *Ae. aegypti* from Pune, despite having considerable viral reads (276,367). WSLV4 was the most frequently occurring unclassified virus found among all the viromes, followed by HMV2. The remaining viruses were specific to either *Ae. aegypti* (Hubei toti-like virus-10, Pacmanvirus A23, Shuangao insect virus-7 (SAIV7), Wenzhou hepe-like virus-1 and *Drosophila immigrans* Nora virus) or *Ae. albopictus* (Wenzhou shrimp virus-9, Wilkie partiti-like virus-2, Hubei picorna-like virus-34 and Hubei sobemo-like virus-9). This shows that distinct viromes of unclassified viruses were found in this study and could possibly be due to host ranges limited to the respective mosquito species. HMV2 had more reads in both the *Ae. albopictus* viromes than the Dibrugarh *Ae. aegypti* virome. However, BLAST results for HMV2 sequences generated from *Ae. albopictus* metagenomics data were confounding. Reads matching to WSLV4 were numerous in all the viromes except for Patiala mosquitoes but with a large variation for each virome (in the order of 10^2^, 10^3^, 10^4^, and 10^5^ for Jodhpur and Dibrugarh *Ae. aegypti* and Kerala and Pune *Ae. Albopictus*, respectively). It is possible that the number of total viral reads or cumulative classified reads may be an influencing factor for the numbers of virus species or family-specific reads.

### 3.7. Phylogenetic Analysis of Virus Sequences Derived from Aedes Viromes

**Figure 7:**
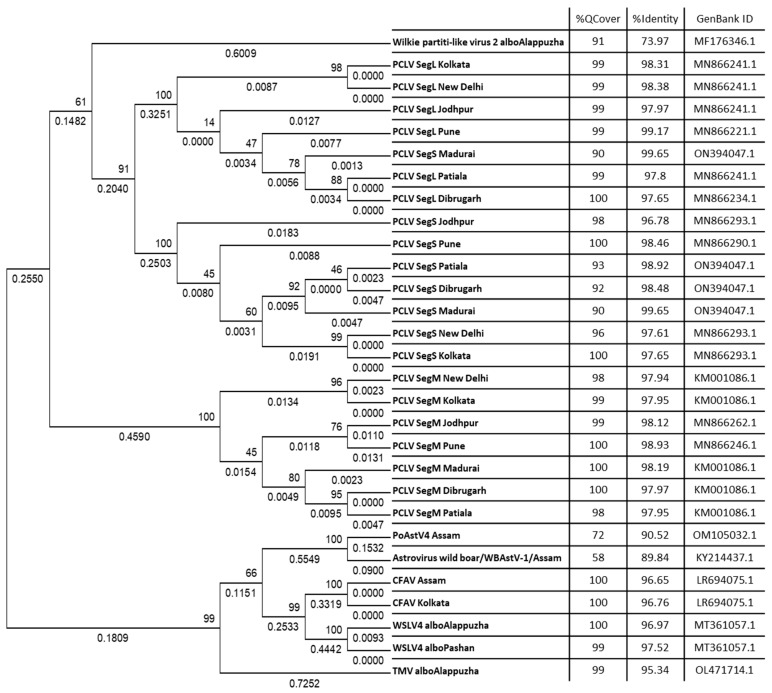
Maximum Likelihood phylogenetic trees: The tree is displayed in the topology-only mode, and construction was performed using the Tamura 3-Parameter model with 1000 bootstrap replicates on the MEGA v.5.2 software. Values below branches indicate the number of substitutions per site, and values at nodes represent the bootstrap support. Each sequence was obtained from corresponding raw data using the Genome Detective online platform, and BLAST searches were performed to find the GenBank nucleotide sequences having the highest percent nucleotide identity with the highest possible query cover (albo: albopictus, PCLV: Phasi Charoen-like phasivirus, Seg: segment, L: large, M: medium, S: small, PoAstV: Porcine astrovirus, WBAstV: Wild boar astrovirus, TMV: Tobacco mosaic virus, CFAV: Cell fusing agent virus, WSLV: Wenzhou sobemo-like virus).

Raw metagenomics data in the form of merged FASTQ files were uploaded onto the Genome Detective online server to generate consensus sequences. Viral genomic sequences or sequences of genomic segments that gave a coverage of >50% were used for phylogenetic analysis. These sequences were processed for BLAST search to find the highest PNI hits. Some FASTA sequences showed ambiguous results through BLAST searches, e.g., they showed high PNI values with viruses of distantly related taxa but with low query coverage. Some sequences also showed comparably high PNIs with more than one taxon at the species level, which further added to the ambiguity toward species specificity. These sequences were subsequently excluded, and the remaining ones were used for phylogenetic analyses (Figure 7). For that, sequences were aligned using MAFFT, and the alignment was used to infer the best DNA nucleotide substitution model that could be used to construct a phylogenetic tree. Tamura 3-Parameter was found to be the best-fit model and hence used to construct Maximum Likelihood (ML) phylogenetic trees with 1000 bootstrap replicates. The alignment was also used to construct a Neighbour-Joining (NJ) phylogenetic tree, resulting in the same clade arrangement as the respective ML trees.

The L, M, and S segments formed three distinct clades (Figure 7). There appeared to be no subclades except for the repeated clustering together of sequences from New Delhi and Kolkata. The M and L segment sequences yielded query coverage of 98–100%, whereas the S segment had a slightly lower coverage, ranging from 90% to 100%. PCLV sequences from Kolkata and New Delhi appeared to have some distinct evolutionary characteristics. The host range of PCLV being limited only to *Ae. aegypti* is also supported by data presented in our study (Figure 3). PCLV formed a distinct clade from all the other viral consensus FASTA sequences analyzed in this study (Figure 7), with the sole exception of Wilkie partiti-like virus-2. Notably, Wilkie partiti-like virus-2 sequence from Kerala *Ae. albopictus* mosquitoes clustered along with the PCLV L and M sequences. However, a paired BLAST of both sequences gave no significant similarity, even with the least stringent parameters. Global Alignment using the Needleman–Wunsch algorithm also yielded only 50% identity [19]. It showed a high query coverage of 91% but a relatively low PNI (73.97%) with GenBank reference sequence MF176346.1 (Wilkie partiti-like virus-2 strain mosWSCP53020, complete genome).

Distinct clades were also formed by WSLV4 and CFAV, as well as TMV and the astroviruses PoAstV4 and WBAstV. Notably, despite constituting only 0.056% of the total viral reads in the virome of *Ae. albopictus* from Kerala, a nearly complete genome of TMV could be generated, indicating the possibility of the presence of infectious virions in mosquitoes.

### 3.8. PCLV Sequences Form Two Distinct Clades Separated by Location and Time

**Figure 8:**
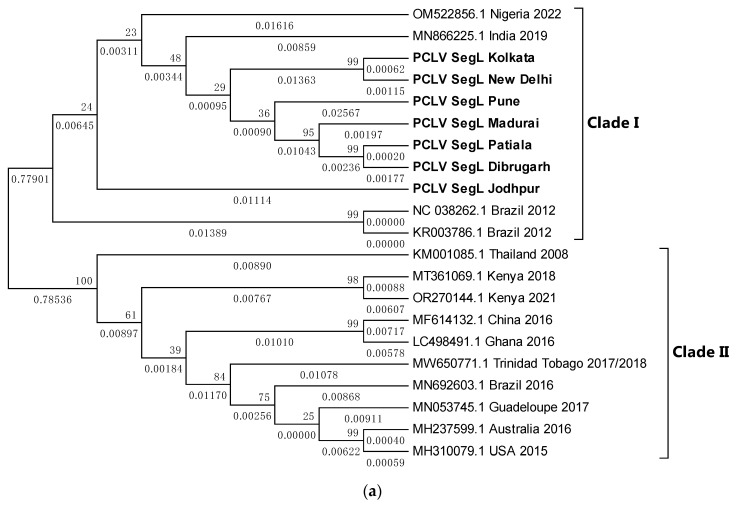

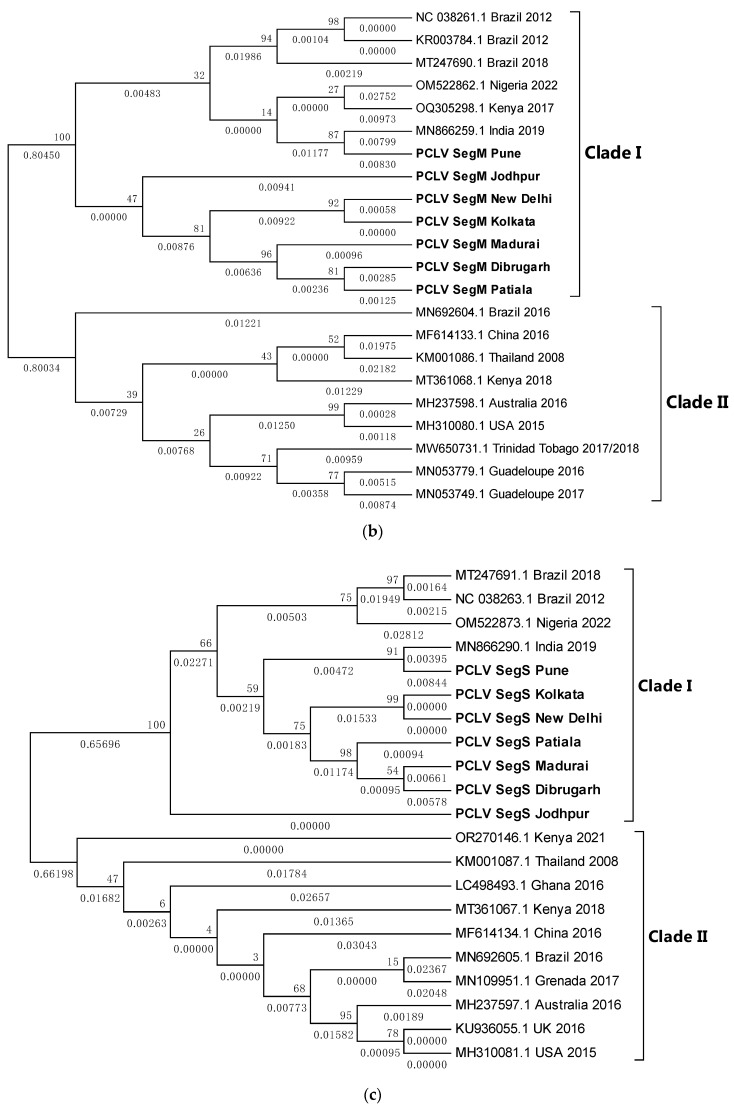
(**a**) Maximum Likelihood phylogenetic tree of Phasi Charoen-like phasivirus segment L sequences: The tree is displayed in the topology-only format and was constructed using the Tamura Nei model, with 1000 bootstrap replicates on the MEGA v.5.2 software. Values below branches indicate the number of substitutions per site, and the values at nodes represent the bootstrap support. Names of countries and years of sample collection/sequence submission are mentioned along with GenBank accession numbers for reference sequences. (**b**) Maximum Likelihood phylogenetic tree of PCLV segment M sequences: The tree is displayed in the topology-only format and was constructed using the General Time Reversible model, with 1000 bootstrap replicates, on the MEGA v.5.2 software. Values below branches indicate the number of substitutions per site, and the values at nodes represent the bootstrap support. Names of countries and years of sample collection/sequence submission are mentioned along with GenBank accession numbers for reference sequences. (**c**) Maximum Likelihood phylogenetic tree of PCLV segment S sequences: The tree is displayed in the topology-only format and was constructed using the Tamura 3-parameter, with 1000 bootstrap replicates, on the MEGA v.5.2 software. Values below branches indicate the number of substitutions per site, and the values at nodes represent the bootstrap support. Names of countries and years of sample collection/sequence submission are mentioned along with GenBank accession numbers for reference sequences.

Overall, PCLV L, M, and S segment sequences formed two distinct clades. Indian PCLV sequences clustered together in a single clade, which also included sequences from Nigeria (for the L segment), Nigeria, Brazil, and Kenya (for the M segment), and Nigeria and Brazil (for the S segment). Clade II of L segment sequences included sequences from Thailand, Kenya, China, Ghana, Trinidad and Tobago, Guadeloupe, Australia, the USA, and Brazil (2016) (Figure 8a); of M segment sequences (Figure 8b) included Brazil, China, Thailand, Kenya, Australia, the USA, Trinidad and Tobago, and Guadeloupe; and of S segment sequences (Figure 8c) included Kenya, Thailand, Ghana, China, Brazil, Grenada, Australia, the United Kingdom, and the USA.

Despite the MEGA software deducing three different models for the construction of the three different PCLV segments’ phylogenetic trees, the clade formation remained constant throughout. The Jodhpur sequence clustered separately from other Indian sequences and formed its own distinct subclade within clade I. The 2021 sequence from Kenya showed the least evolutionary distance (ED) from the common root of all the S segment sequences, and this could have implied that there is no temporal distribution of PCLV S segment mutation and evolution throughout the world. Instead, we see the three segments with independent evolutionary characteristics, with the L segment seemingly following a temporal increase of evolutionary divergence, while the M and S segments apparently do not follow such a trend. The sequences from Brazil 2018 (MT247691.1) and Brazil 2012 clustered together in clade I, whereas the Brazil 2016 sequence (MN692605.1) clustered in clade II.

### 3.9. TMV Sequence from Ae. albopictus Is Closely Related to the South African TMV Sequence

**Figure 9:**
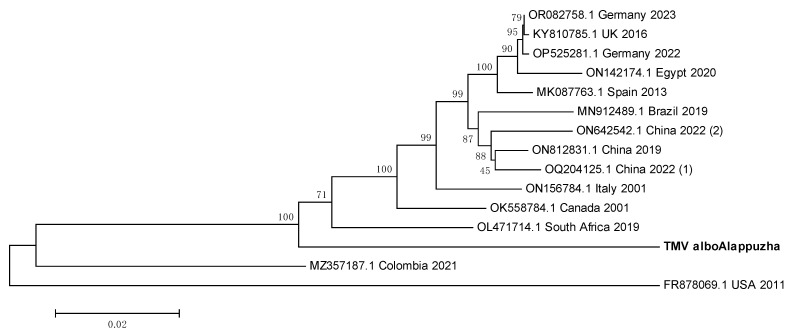
Maximum Likelihood phylogenetic tree of the Tobacco mosaic virus genomic sequence: The tree was constructed using the General Time Reversible model, with 1000 bootstrap replicates, on the MEGA v.5.2 software. Values below branches indicate the number of substitutions per site, and the values at nodes represent the bootstrap support. Names of countries and years of sample collection/sequence submission are mentioned along with GenBank accession numbers for reference sequences.

The whole genome sequence of TMV from Kerala *Ae. albopictus* mosquitoes was used to construct a phylogenetic tree (Figure 9). The Indian sequence seemed to have the least ED with the South African sequence from 2019 (OL471714.1) and may thus be the most evolutionarily related to it.

### 3.10. WBAstV from Belgium Is the Likely Introduction Source of the Virus into Assam

**Figure 10:**
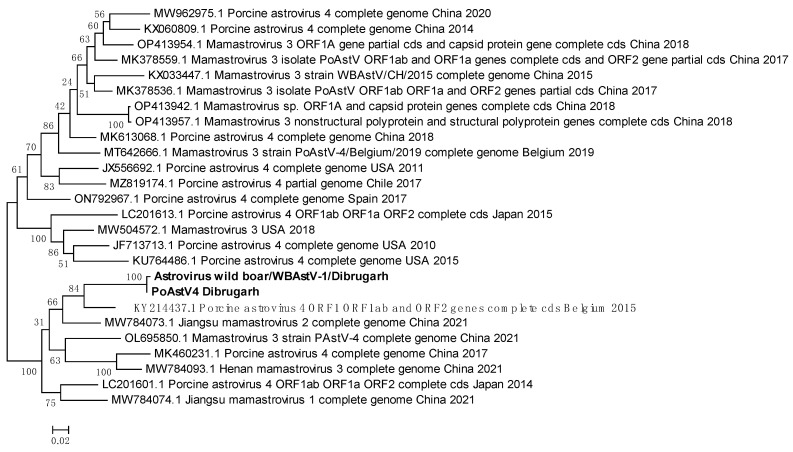
Maximum Likelihood phylogenetic tree of the Porcine astrovirus-4 and Wild Boar astrovirus genomic sequences: The tree is displayed in the topology-only format and was constructed using Tamura 3-parameter, with 1000 bootstrap replicates on MEGA v.5.2 software. Values at nodes represent the bootstrap support. The scale represents evolutionary distance in terms of base substitutions per site. Names of countries from which the samples were collected, and the year of sample collection (or sequence submission) are mentioned, along with the GenBank accession number for each reference. ORF: open reading frame, cds: coding DNA sequence, PoAstV/PAstV: porcine astrovirus, WBAstV: wild boar astrovirus, sp.: species.

It was possible to generate consensus FASTA sequences of the two astroviruses, PoAstV4 and WBAstV, with genome coverages of 72% and 58%, respectively (Figure 7). These showed varying PNIs with multiple sequences of various lengths from multiple astroviruses. Therefore, all the sequences with the highest PNIs found through BLAST were downloaded, and a phylogenetic tree was constructed (Figure 10). The WBAstV sequence showed the highest PNI with a PoAstV4 sequence from Belgium 2015 (KY214437.1) (Figure 7). PoAstV4 and WBAstV sequences from Dibrugarh clustered together with a 99.58% PNI and a query cover of 28% when run on a paired BLAST. This indicates that the WBAstV, as classified by EPI2ME and Genome Detective, belongs to the same taxon as the Porcine Astrovirus and was closest to the Belgium 2015 sequence of PAstV4.

### 3.11. Wilkie Partiti-like Virus- 2 Is Related to the Kuusamo Alphapartitivirus

**Figure 11:**
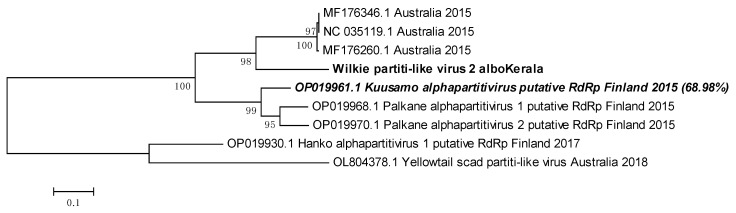
Maximum Likelihood phylogenetic tree of the Wilkie partiti-like virus-2 genomic sequence: The tree was constructed using the Tamura 3-parameter, with 1000 bootstrap replicates, on the MEGA v.5.2 software. Values at nodes represent the bootstrap support. The scale represents evolutionary distance in terms of base substitutions per site. Names of countries from which the samples were collected, and the year of sample collection/sequence submission are mentioned, along with the GenBank accession number for each reference.

Wilkie partiti-like virus-2 genomic sequence with 91% coverage was obtained from Kerala *Ae. albopictus* mosquitoes. Since the virus is unclassified, we used the next most identical nucleotide sequences from viruses as found through BLAST for phylogenetic tree building (Figure 11). PNIs of our sequence with already available Wilkie partiti-like virus-2 sequences were relatively low, with none above 73.97% in the phylogenetic tree analysis. Wilkie partiti-like virus-2 sequence from Kerala *Ae. albopictus* clustered together with three other sequences in the same clade. The other viruses used in the analysis were found using the Discontiguous Megablast option in the BLAST suite. Palkane alphapartitivirus-2 (OP019970.1) and Kuusamo alphapartitivirus (OP019961.1) had similar PNIs as the Kerala virus sequence (68.99% and 68.98%, respectively). Nevertheless, it was difficult to draw a meaningful conclusion regarding the taxonomic placement of this virus.

### 3.12. WSLV4 Belongs to Either the Solemoviridae Family or the Tolivirales Order

**Figure 12:**
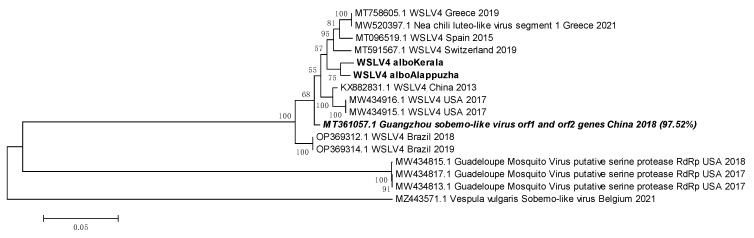
Maximum Likelihood phylogenetic tree of Wenzhou Sobemo-like virus-4 genomic sequences: The tree was constructed using the Tamura Nei model, with 1000 bootstrap replicates, on the MEGA v.5.2 software. Values at nodes represent the bootstrap support. The scale represents evolutionary distance in terms of base substitutions per site. Names of countries from which the samples were collected, and the year of sample collection (or sequence submission) are mentioned, along with the GenBank accession number for each reference. orf: open reading frame, albo: albopictus, RdRp: RNA dependent RNA polymerase.

WSLV4 sequences from *Ae. albopictus* mosquitoes of Kerala and Pune were used to construct a phylogenetic tree (Figure 12). The Indian sequences clustered tightly. The least ED was shown with a sequence from China, 2013 (KX882831.1). China has the closest geographic proximity to India out of all the source locations of the WSLV4 reference sequences in the tree. At the same time, Indian WSLV4 sequences showed close evolutionary relationships and low EDs with existing WSLV4 sequences, and sequences from two other viruses, the Nea chili luteo-like virus and the Guangzhou sobemo-like virus, also clustered with the WSLV4 sequences. This made the deduction of possible taxonomic placement of WSLV4 ambiguous. BLAST analysis of the sequence derived from Pune *Ae. albopictus* mosquitoes showed PNIs of 97.52% with the Guangzhou sobemo-like virus and 97.24% with the Nea chili luteo-like virus. Despite the PNI difference being marginal, we may infer that the WSLV4 is more closely related to the Guangzhou sobemo-like virus, which belongs to the family *Solemoviridae*, and the Nea chili luteo-like virus, belonging to order *Tolivirales*. While this is an inference drawn from nucleotide sequences, more investigation based on morphology, ultrastructure, and antigenicity is needed for confirmation.

### 3.13. Indian CFAV Sequences Clustered Together to Form a Single Clade

**Figure 13:**
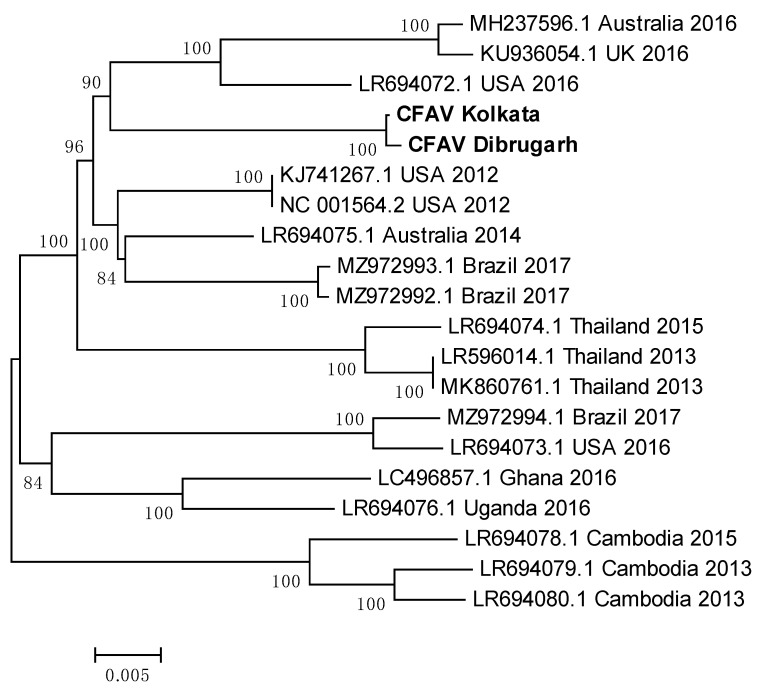
Maximum Likelihood phylogenetic tree of Cell fusing agent virus: genomic sequences: The tree was constructed using the General Time Reversible model, with 1000 bootstrap replicates, on the MEGA v.5.2 software. Values at nodes represent the bootstrap support. The scale represents evolutionary distance in terms of base substitutions per site. Names of countries from which the samples were collected, and the year of sample collection (or sequence submission) are mentioned, along with the GenBank accession number for each reference.

CFAV sequences from *Ae. aegypti* mosquitoes of Dibrugarh and Kolkata were used to construct a phylogenetic tree with CFAV whole genome reference sequences from across the world (Figure 13). A list of PNIs of Indian CFAV sequences with reference sequences from across the world is given in Table 4. Sequences from Dibrugarh and Kolkata had a PNI of 99.88%, indicating their close relationship. Australia 2014 reference sequence (LR694075.1) showed the highest PNIs with the Indian sequences, suggestive of an introduction from *Ae. aegypti* mosquitoes of Australia or vice versa. The least PNI was noted with a 2015 sequence from Cambodia (LR694078.1). Despite these two reference sequences being from samples collected only one year apart, they have low evolutionary relatedness, indicating that the location of a mosquito population may decide the characteristics of a CFAV population. Sequences from Cambodia (2013 and 2015) and Thailand clustered together, implying that the evolution of the virus is slow (Figure 13). Sequences from Brazil (2017) and the USA (2016) grouped in the same clade, indicating a minimal ED between the two, possibly due to the circulation of the same virus. It may be interesting to analyze CFAV sequences available from Mexico to check for any spatial relationship. Interestingly, sequences from Ghana and Uganda clustered together to form an independent clade despite being on the western and eastern sides of Africa.

## 4. Discussion

We report the metaviromic characterization of *Ae. aegypti* mosquitoes from seven geographically diverse locations of India and *Ae. albopictus* mosquitoes from two geographically distinct regions. Of these, one location, Pune, Maharashtra, had mosquitoes of both the species analyzed, thus allowing for comparison of viromes between the two prominent anthropophilic *Aedes* species. We employed NGS using the ONT platform, taking advantage of the long reads generated through Nanopore sequencing. The aim was to identify viruses inhabiting mosquito populations and try finding the known human pathogenic viruses, which makes this, to our knowledge, the first report of its kind from India.

Akin to our previous report [13], a large proportion of the reads was assigned to *Homo sapiens*, ranging from 4566 reads to 167,340 reads across all the viromes. In the previous study, we used the Centrifuge v1.0.4 database for metagenomic taxa assignment, whereas in this one, the EPI2ME Portal’s classification system (Kraken2) was used. A report from 2018 elucidates the occurrence of high numbers of *H. sapiens* reads obtained from RNA sequencing (RNA-seq) data of mosquitoes fed on blood meals [20]. Some reads also matched to DNA from mice/rats, cows, and dogs, implying that blood meals can introduce RNA and DNA from hosts to mosquitoes sampled for metagenomics. In fact, Avian endogenous retrovirus EAV-HP was found to be integrated into the genomes of *Gallus* spp. (chicken) in a non-random manner, as evidenced by Sequence Read Archive (SRA) dataset analyses [21] and full genomes sequenced from the whole blood of village chickens of Ethiopia [22]. We detected EAV-HP in the virome of Patiala mosquitoes. While DNase and RNaseI treatment was performed for all homogenized mosquito samples before viral RNA extraction, these findings indicated that the treatment might have been insufficient. Furthermore, the low genomic coverage of CfGV, i.e., DNA viruses with large genomes, implies that they may not be well-represented in the metaviromics data we generated, and our protocol has worked better on RNA viruses.

Detection of *Serratia* phage Muldoon, which infects the bacterium *Serratia marcescens*, in Madurai *Ae. aegypti* led us to look for *S. marcescens* reads. A total of 7097 reads assigned to *S. marcescens* was found in Madurai data, in addition to 7572 reads from Patiala, 1124 reads from Dibrugarh, and 877 reads from Jodhpur. This was coupled with the presence of *Serratia* phage Muldoon in the Madurai virome, which strongly indicated the presence of *S. marcescens* in the mosquitoes sampled. This bacterium is known to influence the modulation of midgut genes in mosquitoes and inhibit the development of the parasite *Plasmodium berghei*[23]. Importantly, *S. marcescens* has been reported to act as a commensal in *Ae. aegypti* and enhance vector competence for DENV [24]. We did not detect any medically important arboviruses in any of our viromes. It would be worthwhile to study the presence of *S. marcescens* in mosquitoes and the vector competence of Indian mosquitoes, as *S. marcescens* not only causes opportunistic nosocomial infections in humans [25] but also possesses resistance mechanisms to antibiotics. Reports of *S. marcescens* infection were reported from Punjab, and its source was found to have been a soap dispenser in the Intensive Care Unit (ICU) [26]. Similarly, an outbreak of *S. marcescens* was reported in a tertiary care hospital in Chennai, Tamil Nadu, near Madurai in 2019 [27]. *Anopheles* spp. mosquitoes can also transmit *S. marcescens* and *P. berghei* through their saliva [28]. This raises the question of whether the *Ae. aegypti* of Madurai, which seemingly harbored *S. marcescens*, can transmit it to humans.

The Dibrugarh mosquito virome had the highest percentage (~12%) of reads matching unclassified viruses, amongst a total of 38,089 viral reads, while the virome of Pune *Ae. aegypti* mosquitoes lacked any reads matching unclassified viruses despite having relatively high viral reads (276,367) (Table 2). In contrast, Pune *Ae. albopictus* virome harbored the highest percentage of unclassified virus reads (~97%) with total viral reads of 122,369. This makes the absence of unclassified virus reads in Pune *Ae. aegypti* virome is an exceptional occurrence and warrants further investigation.

Our observations include a trend of increasing viral read counts with increasing cumulative (Eukaryota, Viruses, Bacteria, and Archaea) read counts. When ONT sequencing and the EPI2ME portal, coupled with the usage of a Taq-based polymerase, was used for testing the read assignment efficiency of samples spiked with Bovine herpesvirus 1 (BoHV-1), there appeared to be a positive correlation between the number of amplification cycles, cumulative read counts, and BoHV-1 specific reads [29]. We used SISPA mediated by Taq polymerase, and thus, a similar occurrence may have happened here, as observed by the vastly differing WSLV4 read numbers for Jodhpur and Dibrugarh *Ae. aegypti* viromes, and Pune and Kerala *Ae. albopictus* viromes. A report from 2015 describes how varying viral titers resulted in varying read numbers for different viruses such as Ebola, Hepatitis C, and CHIKV [30]. Another report mentions the correlation between initial viral load and the number of reads mapped to the Influenza A H1N1 virus [31]. Viral load dependence of read numbers for virus spiking experiments has also been reported in yet another study [32]. These studies point towards the correlation of viral load with read numbers and can thus be used to explain multiple observations in our study that hint at this relationship. We have, however, noted a variation in the number of PCLV L, M, and S segment-specific reads from the same samples. This could be probably due to either replication intermediates contributing as templates or virions not necessarily having encapsidated one molecule of each segment [33].

The question of whether long-read sequencing has an advantage over short-read sequencing arises. Indeed, a comparative analysis of both kinds of sequencing has shown that homologs that are more distant may not be detected by short-read sequencing, i.e., when read lengths are between 100–200 bp [34]. Sequencing of relatively small reads may lead to incorrect taxon assignments. Whether such a phenomenon has occurred in our experiments and analyses is unclear, but the ambiguous results of sequences run on BLAST become more explicable if viewed through this lens. We found multiple consensus FASTA sequences generated through Genome Detective and assigned independently by the EPI2ME portal, giving comparably high nucleotide identities to multiple taxa. Such a confounding occurrence may be explained by the still relatively short read length that resulted from the SISPA-generated amplicons in our experiments.

Another observation was the occurrence of porcine astroviruses PoAstV4 and WBAstV in the virome of Dibrugarh mosquitoes. Detection of a porcine virus in mosquitoes will not be the first, as there is experimental evidence of mechanical transmission of porcine reproductive and respiratory syndrome virus from infected pigs to naïve ones through *Aedes vexans* mosquitoes [35]. Similarly, Porcine circovirus-2 has been detected in mosquitoes from pig farms in the Hubei and Anhui provinces of China [36]. Thus, it is likely for PoAstV4 and WBAstV to occur in mosquitoes collected from Dibrugarh, though there are no reports of porcine astrovirus infections in Assam. Another possibility could be the association of the virus with mosquito larval or pupal stages, which thrive in aquatic environments infested with enteric viruses such as adenovirus and circovirus [37]. Importantly, these findings indicate the possibility of porcine astrovirus circulation in Assam.

Sequences of all the three segments of PCLV were generated from *Ae. aegypti* samples of all locations. Phylogenetic trees displayed a divergence of sequences into two distinct clades: clade-I and clade-II. PCLV appears to have undergone different evolutionary processes for its three segments, as evidenced by varying placements of the sequences from the same location but from different time periods in different clades. This implies that PCLV may experience events similar to the Influenza virus or Rotavirus, where in the multi-segmented genomes are mixed and assembled when two strains infect the same host. PCLV is an ISV that solely infects *Ae. aegypti*, with no occurrence in *Ae. albopictus* reported so far. Attempts to isolate the virus in our laboratory from PCR-positive mosquito samples using the *Ae. albopictus* C6/36 cell lines were unsuccessful (unpublished data). Thus, seemingly inexplicable phylogenetic characteristics may be explained by considering the possibility of exchanges of PCLV genomic segments when *Ae. aegypti* from different locations mate. Whether this really happens in nature or not needs confirmation using in vivo experiments. The evolutionary trend of PCLV may also be correlated with the spread of *Ae. aegypti* throughout the world. Publications have taken into account both mosquito-transmitted virus outbreak history and population genomics to guess at the probable historical spread of *Ae. aegypti* [38]. To correlate both the phenomena, i.e., the spread of *Ae. aegypti* and that of PCLV, knowledge of the origin of PCLV will also be required.

*Ae. aegypti* is a highly anthropophilic mosquito and *Ae. albopictus* is only second to it as a major vector for arboviruses. This human-centered behavior of *Ae. aegypti* arose initially due to climate change and, as of the past 20–40 years, due to rapid urbanization [39]. As the mosquitoes started becoming more anthropophilic, a higher emergence of arboviral disease spread was observed. The role of this, if played, in the global spread of CFAV needs to be investigated. In this study, we found that Indian CFAV sequences are almost identical to a 2015 Australian CFAV sequence. Interestingly, there is evidence of CFAV integration into *Ae. albopictus* genome. The integrated segments exist as nonretroviral endogenous viral elements (nrEVEs) and contain intact viral protein-coding regions [40]. These are speculated to be remnants of the virus that had infected and settled into *Ae. albopictus* populations a long time ago and has since integrated itself into its genome. However, CFAV was not found in both the *Ae. albopictus* viromes in the present study. Genomic characteristics of CFAV may thus be interesting to study and could shed light on its possible influence on arboviral pathogens.

Our study also had a few drawbacks. Despite numerous reads having been mapped to certain viruses, for example, the CfGV, there was poor genomic coverage, and thus, whether the viable, infectious virions of the virus are present in the mosquitoes remains unclear. The reads generated by our protocol were on an average of ~476 bases, and although longer than that generated through short-read technologies, they may not be informative or specific enough to correctly assign taxa. Due to the long length of eukaryotic or prokaryotic genes and the presence of introns in the former, there is a chance of erroneous assignments occurring due to relatively short reads. One report mentions how the EPI2ME pipeline made use of poor-quality bacterial and viral genome assemblies available on RefSeq, which led to the incorrect assignment of BoHV-1 sequences to other viral and bacterial taxa [29].

In conclusion, we were able to discover viruses circulating in mosquito populations from geographically diverse sampling locations across India, and through this, we were able to detect certain viruses that have implications for major pathogens. Circumstantial evidence generated by other studies on the probability of *S. marcescens* circulation in mosquitoes of Tamil Nadu (and the possibility of human infections resulting from it), the experimental transmission of porcine viruses through mosquitoes, and the valid presence of the CFAV in mosquitoes of Assam and West Bengal, has relevance in terms of animal and human diseases. This is the first report of countrywide viral surveillance through the use of untargeted metagenomics from India, and these results may lead to further studies to explain the influence of these viruses on the replication and transmission of viruses of clinical importance.

## Figures and Tables

**Table 1:** Details of primers used for SISPA and CFAV capsid region amplification.

Primer Name	Sequence	Reference
Random anchored reverse primer RT5	CATCACATAGGCGTCCGCTGNNNNNN	[12]
Anchored forward primer P5	CATCACATAGGCGTCCGCTG	
Random anchored reverse primer RT12	GGTGGGCGTGTGAAATCGACNNNNNN	
Anchored forward primer P12	GGTGGGCGTGTGAAATCGAC	
Random anchored reverse primer IDT-K-8N	GACCATCTAGCGACCTCCACNNNNNNNN	[14]
Anchored forward primer IDT-K	GACCATCTAGCGACCTCCAC	
CFAV-Capsid-32F	CAGTTTGGGTCACGCTTA	This study
CFAV-Capsid-536R	ATGTCAATCACCACGCAT	

**Table 2:** Quality scores and classification of the metagenomics data for *Ae. aegypti* mosquitoes.

(**a**)							
**Sampling Location**	**Average Quality Score**	**Average Sequence Length (Bases)**	**Reads Analyzed**	**Reads Classified**	**Reads Unclassified**	**Cumulative Reads (Eukaryota, Viruses, Bacteria and Archaea)**	**Virus Reads**
Dibrugarh	12.29	378	2,566,367	1,148,796	1,409,637	1,097,121	38,089
Jodhpur	12.40	357	1,416,476	605,659	805,246	604,734	35,220
Kolkata	12.29	318	1,258,839	246,022	1,008,574	214,756	52,798
Madurai	11.91	286	7,786,762	3,749,144	3,997,735	2,945,046	808,370
New Delhi	12.41	280	1,728,868	761,242	960,985	595,398	140,179
Patiala	12.48	300	5,966,644	3,494,089	2,472,555	3,414,206	188,311
Pune	12.67	401	2,415,355	774,139	344,458	612,756	276,367
(**b**)							
**Sampling Location**	**Average Quality Score**	**Average Sequence Length (Bases)**	**Reads Analyzed**	**Reads Classified**	**Reads Unclassified**	**Cumulative Reads (Eukaryota, Viruses, Bacteria and Archaea)**	**Total Virus Reads**
Pune	11.27	476	359,883	153,303	204,490	153,303	122,369
Alappuzha	10.75	296	514,027	185,691	306,661	185,691	61,982

**Table 3:** Virus species and reads in *Ae. aegypti* viromes from different locations.

(a)							
**Location**	**Patiala (Punjab)**	**Pune (Maharashtra)**	**Dibrugarh (Assam)**	**Madurai (Tamil Nadu)**	**New Delhi**	**Jodhpur (Rajasthan)**	**Kolkata (West Bengal)**
Species identified 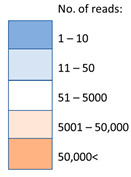	Phasi Charoen-like phasivirus	Phasi Charoen-like phasivirus	Phasi Charoen-like phasivirus	Phasi Charoen-like phasivirus	Phasi Charoen-like phasivirus	Phasi Charoen-like phasivirus	Phasi Charoen-like phasivirus
	*Choristoneura fumiferana* granulovirus	*Choristoneura fumiferana* granulovirus	Cell fusing agent virus	*Choristoneura fumiferana* granulovirus	*Choristoneura fumiferana* granulovirus	Wenzhou sobemo-like virus 4	Cell fusing agent virus
	Cell fusing agent virus	Shamonda orthobunyavirus	Wenzhou sobemo-like virus 4	Shamonda orthobunyavirus	Cell fusing agent virus	*Choristoneura fumiferana* granulovirus	*Choristoneura fumiferana* granulovirus
	Avianendogenous retrovirus EAV-HP	*Lactobacillus* virus LP65	*Choristoneura fumiferana* granulovirus	Hubei toti-like virus 10	Shamonda orthobunyavirus	Tobacco mild green mosaic virus	Ilheus virus
	*Ostreococcus lucimarinus* virus 1	Cyprinid herpesvirus 2	Hubei mosquito virus 2	Badu phasivirus	*Lactobacillus* virus LP65	Aroa virus	Tobacco mosaic virus
	*Lactobacillus* virus LP65	Ostreid herpesvirus 1	Porcine astrovirus 4	*Serratia* phage Muldoon	Wenzhou hepe-like virus 1	Ilheus virus	Aroa virus
	*Chrysochromulina ericina* virus	*Bacillus* virus G	Astrovirus wild boar/WBAstV-1/2011/HUN	*Synechococcus* phage S-CAM4	*Drosophila immigrans* Nora virus	*Culex* Flavi-like virus	Shamonda orthobunyavirus
	*Cotesia congregata* bracovirus	*Lactobacillus* virus LLKu	Ilheus virus	*Salinivibrio* phage CW02	*Sphingomonas* phage PAU	*Synechococcus* phage S-CAM22	Shuangao insect virus 7
	*Lactobacillus* virus LLKu	Chilli ringspot virus	Astrovirus MLB2	*Mycobacterium* phage UnionJack	Esparto virus	*Chrysochromulina ericina* virus	
	Badu phasivirus	Aotine betaherpesvirus 1	Shamonda orthobunyavirus	*Jacquemontia* yellow vein virus	*Trichoplusia ni* ascovirus 2c		
	Wenzhou sobemo-like virus 4	*Tenacibaculum* phage PTm1	Porcine astrovirus 2	Esparto virus	Golden Marseillevirus		
	*Euproctis pseudoconspersa* nucleopolyhedrovirus	Lactococcus virus KSY1	*Agrotis ipsilon* multiple nucleopolyhedroviruses	*Cotesia congregata* bracovirus			
	*Bacillus* virus G	*Vibrio* phage douglas 12A4	Cyprinid herpesvirus 2				
	*Rhinolophus* associated gemykibivirus 2	*Cotesia congregata* bracovirus					
	Pacmanvirus A23	*Acanthamoeba polyphaga* mimivirus					
	*Pandoravirus quercus*	*Amsacta moorei* entomopoxvirus					
	Shamonda orthobunyavirus	*Pandoravirus neocaledonia*					
	*Emiliania huxleyi* virus 86						
(b)							
**Location**		**Alappuzha (Kerala)**			**Pune (Maharashtra)**		
Species Identified 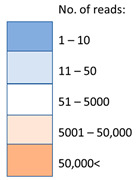		Wenzhou sobemo-like virus 4			Wenzhou sobemo-like virus 4		
		*Choristoneura fumiferana* granulovirus			Hubei mosquito virus 2		
		Hubei mosquito virus 2			*Choristoneura fumiferana* granulovirus		
		Tobacco mosaic virus			Wenzhou shrimp virus 9		
		Shamonda orthobunyavirus			Shamonda orthobunyavirus		
		Cyprinid herpesvirus 2			*Mythimna unipuncta* nucleopolyhedrovirus		
		Elephant endotheliotropic herpesvirus 4					
		Wilkie partiti-like virus 2					
		Tobacco mild green mosaic virus					
		Tomato brown rugose fruit virus					
		Tomato mottle mosaic virus					
		Hubei picorna-like virus 34					
		Hubei sobemo-like virus 9					
		African swine fever virus					
		*Pandoravirus quercus*					

**Table 4:** Percent nucleotide identities of CFAV whole genome sequences obtained in this study with reference genomes from across the world. Values in green indicate the highest Percent Nucleotide Identities, whereas values in red indicate the lowest Percent Nucleotide Identities.

Sequence/Reference	Country	Year of Sample Collection/Sequence Submission	CFAV Kolkata	CFAV Dibrugarh
CFAV Kolkata	India	2022	100	99.88
CFAV Dibrugarh			99.88	100
LR694075.1	Australia	2014	96.75	96.54
KJ741267.1	USA	2012	96.7	96.51
NC 001564.2			96.7	96.51
MZ972993.1	Brazil	2017	96.22	95.98
MZ972992.1			96.22	95.98
MH237596.1	Australia	2016	95.56	95.34
KU936054.1	UK		95.51	95.29
LR694072.1	USA		96.31	96.11
LR596014.1	Thailand	2013	95.41	95.22
MK860761.1			95.41	95.22
LR694074.1		2015	95.35	95.16
LC496857.1	Ghana	2016	95.28	95.04
LR694076.1	Uganda		95.48	95.25
MZ972994.1	Brazil	2017	94.81	94.56
LR694073.1	USA	2016	94.86	94.61
LR694079.1	Cambodia	2013	94.5	94.26
LR694080.1			94.52	94.28
LR694078.1		2015	94.38	94.15

## Data Availability

The generated metagenome data has been submitted to the NCBI BioSample database. Sample accession IDs and respective links are as follows: SAMN39246597 India (Patiala) *Ae. aegypti* 2021-08 mosquito metagenome; SAMN39246598 India (Pune) *Ae. aegypti* 2021-01 mosquito metagenome; SAMN39246599 India (Dibrugarh) *Ae. aegypti* 2020-03 mosquito metagenome; SAMN39246600 India (Madurai) *Ae. aegypti* 2020-04 mosquito metagenome; SAMN39246601 India (New Delhi) *Ae. aegypti* 2021-08 mosquito metagenome; SAMN39246602 India (Jodhpur) *Ae. aegypti* 021-08 mosquito metagenome; SAMN39246603 India (Kolkata) *Ae. aegypti* 2021-08 mosquito metagenome; SAMN39246604 India (Alappuzha, Kerala) *Ae. albopictus* 2023-01 mosquito metagenome; SAMN39246605 India (Pune) *Ae. albopictus* 2022-08 India (Pune) mosquito metagenome; https://www.ncbi.nlm.nih.gov/biosample/39246597; https://www.ncbi.nlm.nih.gov/biosample/39246598; https://www.ncbi.nlm.nih.gov/biosample/39246599; https://www.ncbi.nlm.nih.gov/biosample/39246600; https://www.ncbi.nlm.nih.gov/biosample/39246601; https://www.ncbi.nlm.nih.gov/biosample/39246602; https://www.ncbi.nlm.nih.gov/biosample/39246603; https://www.ncbi.nlm.nih.gov/biosample/39246604; https://www.ncbi.nlm.nih.gov/biosample/39246605 (all accessed on 1 December 2023).
